# Molecular Diagnostics for AIDS Lymphoma Diagnosis in South Africa and the Potential for Other Low- and Middle-Income Countries

**DOI:** 10.1200/JGO.17.00043

**Published:** 2017-08-25

**Authors:** Samantha L. Vogt, Moosa Patel, Tanvier Omar, Sugeshnee Pather, Neil Martinson, Richard Ambinder

**Affiliations:** **Samantha L. Vogt** and **Richard Ambinder**, Johns Hopkins Sidney Kimmel Comprehensive Cancer Center, Baltimore, MD; **Moosa Patel**, **Tanvier Omar**, and **Sugeshnee Pather**, University of the Witwatersrand, Johannesburg; and **Neil Martinson**, Perinatal HIV Research Unit, Soweto, South Africa.

In 2015, the HIV prevalence in adults 15 to 49 years of age in South Africa was 19.2%, which represented an estimated 7 million HIV-infected individuals.^1^ In 2004, the government of South Africa started its public health antiretroviral therapy (ART) program^2^ and during the past 12 years, has initiated ART for more than 3 million people.^3^ Coincident with this, the overall prevalence of HIV-associated lymphomas has increased during the past decade.^4,5^ There are many possible reasons for this increase despite increased ART coverage, such as late initiation of ART therapy, incomplete ART coverage, and perhaps a long enough lifespan for some patients to develop lymphoma.

HIV infection is associated with an increased risk of lymphomas.^6-9^ The most common subtypes diagnosed in people living with HIV (PLWH) are diffuse large B-cell lymphoma, Burkitt’s lymphoma, and Hodgkin lymphoma (HL).^10^ In South Africa, plasmablastic and non-Hodgkin lymphoma (NHL) with intermediate features between diffuse large B-cell lymphoma and Burkitt’s lymphoma are being increasingly recognized in PLWH.^4^ Clinical signs and symptoms of aggressive lymphoma often are indistinguishable from common opportunistic infections, the most notable of which is *Mycobacterium tuberculosis* infection (ie, tuberculosis [TB]). According to the WHO Global Tuberculosis Report in 2016, South Africa has the highest burden of HIV and TB coinfection—473 occurrences per 100,000 people annually and an estimated 10,000 occurrences of multidrug-resistant (MDR) TB each year.^11^ In addition, TB is the leading cause of death recorded on death certificates in South Africa^12^ and autopsy studies suggest it is the leading infection found postmortem in HIV-infected adults receiving ART,^13^ in those who die in the hospital,^14^ and in people who die at home without an apparent cause of death.^15^

TB in PLWH often is difficult to confirm by traditional microscopy, because roughly half of HIV-infected patients present with smear-negative disease^16^; those who present with fever, night sweats, weight loss, and lymphadenopathy often are started empirically on TB treatment.^17^ For those patients who do not respond to initial therapy, MDR TB is always a consideration. Empiric treatment of TB can result in major delays in diagnosis and initiation of appropriate treatment. Indeed, a retrospective review conducted in KwaZulu-Natal, South Africa, documented that 18 of 21 patients with lymphoma had been treated empirically for TB for a median of 5 months before the diagnosis of lymphoma was established and they were referred for lymphoma treatment.^18^ In addition, a study from Uganda showed that 30.6% (56 of 183) of patients with HIV-associated lymphoma had received an average of 3.5 months of empiric TB treatment before the lymphoma diagnosis,^19^ which suggests that this problem of possible delayed diagnoses is not isolated to South Africa.

Simply increasing the number of biopsies is not practical given the limited resources available. After biopsy specimens are prepared, an anatomic pathologist is required for interpretation. Minimal training for a pathologist requires 4 years beyond medical school. South African pathologists are already hard pressed to interpret the biopsies that are currently obtained and would struggle to accommodate a large increase in biopsies. Also, the numbers of pathologists in South Africa far exceed those in many low-and middle-income countries (LMICs).^20^

These limitations notwithstanding, increased capacity to diagnose lymphoma may be lifesaving. Studies from developed countries suggest overall long-term survival rates of at least 50% for systemic HIV-associated lymphoma^21-25^ by using standard chemotherapy paradigms in combination with ART. Although limited data exist about the outcomes of HIV-associated lymphoma in South Africa, initial reports suggest that long-term survival can be achieved with combined ART and chemotherapy in approximately half of the patients. Poor performance status, CNS involvement, compromised hepatic or renal function, and bulky disease all adversely affect outcomes and are more common in patients with delayed diagnoses. A mechanism to better prioritize patients for timely diagnostic biopsy likely would pay dividends in lives saved. Rapid advances in molecular diagnostics might facilitate such prioritization.

Antibody diversity is generated by recombination of immunoglobulin DNA loci in B cells. The recombination of various variable, diversity, and joining segments on the heavy-chain locus and the variable and joining segments on the two light-chain loci (κ and λ) in bone marrow create unique immunoglobulin molecules that can leave the bone marrow and travel to peripheral lymphoid organs (spleen, lymph nodes, and mucosa-associated lymphoid tissue). In these peripheral lymphoid organs, additional modification of the immunoglobulin molecule occurs through a process known as somatic hypermutation. Taken together, the various modifications that immunoglobulin molecules undergo increase the diversity of the immunoglobulin repertoire and create B cells that have their own unique signatures.

Lymphoid neoplasms are a clonal expansion of a particular B-cell population. This B-cell population can be detected and characterized by the unique signature of the immunoglobulin molecule present. Southern blot was an early technique used to detect clonal immunoglobulin gene rearrangements as a marker of lymphoma.^26-28^ As rearrangement of the immunoglobulin gene segments alters the position of the restriction-endonuclease sites, a clonal population can be detected by the presence of a distinct band on SB that differs from that of germline sequences.^26-28^ Subsequently, much more sensitive approaches that involve polymerase chain reaction technology with standardized primer sets have been developed.^29-31^

In parallel to progress in the technical approach to detecting clonal immunoglobulin rearrangements, appreciation has grown that tumor DNA often is present in patients’plasma.^32^ Investigation of circulating tumor DNA (ctDNA; a technique often termed liquid biopsy) is actively being studied in many malignancies. Several groups have demonstrated the applicability to lymphoma diagnostics and monitoring, including in patients with AIDS lymphoma.^33-35^

More recently, next-generation sequencing (NGS) has emerged as a precise and efficient way to detect clonal immunoglobulin gene rearrangements in ctDNA; the level of detection is one tumor cell per million leukocytes.^36^ Several studies that use NGS approaches (LymphoSight; Adaptive Technologies, Seattle, WA) have demonstrated an ability to detect clonal immunoglobulin gene rearrangements in the ctDNA of HIV-negative patients with NHL^37-41^ and HL.^42^ Wagner-Johnston et al^4^ reported a sensitivity of 50% (in 24 of 48 samples) for detection of clonal immunoglobulin DNA by NGS in pretreatment samples taken from PLWH with either NHL or HL.

More research is needed to better understand the sensitivity and specificity of NGS of clonal immunoglobulin gene rearrangements in ctDNA from patients with aggressive B-cell lymphomas, including PLWH. Although inadequate alone for lymphoma diagnosis, NGS of ctDNA in blood samples could prioritize biopsies in those whose clinical presentation suggests a high risk of lymphoma. Such a two-stage diagnostic strategy could allow the surgical and histopathologic resources available in low-resource settings to be leveraged so as to maximize the diagnosis of lymphoma–especially when lymphoma diagnoses may be missed or delayed because of the high suspicion of TB or other infection.

Although NGS is not currently available in resource-limited settings for incorporation into the diagnostic algorithm for lymphoma, precedence exists for the introduction of sophisticated diagnostic approaches that use DNA technology in South Africa. The GeneXpert platform developed by Cepheid (Sunnyvale, CA) first emerged for the detection of *Bacillus anthracis* by the US Postal Service.^43^ The system produced rapid results (within 30 to 40 minutes) and because each individual cartridge contains the polymerase chain reaction primers for the target nucleic acid sequence of interest, the technology has been adapted for various infectious and oncologic diagnostic purposes. In South Africa, GeneXpert has replaced microscopy as the first-line diagnostic test for TB and was endorsed by WHO in 2010. GeneXpert machines in South Africa can be loaded by technicians who require only 2 weeks of training beyond basic schooling. Of particular interest, GeneXpert allows for the diagnosis of rifampin-resistant TB.^44^

As alluded to previously, HIV-associated TB can be particularly challenging to diagnose in a timely fashion with smear microscopy and culture. A meta-analysis of seven studies conducted in HIV-positive patients who lived in LMICs evaluated the sensitivity of sputum microscopy compared with that of the GeneXpert TB assay with culture positivity serving as the gold standard. The results showed a median sensitivity with smear microscopy of 52.8% (range, 22.2% to 68.9%) and a median sensitivity with GeneXpert TB of 84.0% (range, 58.3% to 91.7%).^45^ Although the majority of these studies were conducted in symptomatic patients, at least one study evaluated active-case finding in a group of ART-naïve, HIV-infected patients establishing care at an ART clinic in Cape Town, South Africa.^46^

In a relatively short amount of time, the GeneXpert platform was successful in garnering WHO approval, accelerating the diagnosis and treatment of TB in symptomatic patients^47^ and contributing to the diagnosis of at-risk individuals in the form of active-case finding^46^ and to the successful negotiation of decreased costs for LMICs.^48^ With the appropriate international collaboration, we believe that NGS could be similarly successful for lymphoma diagnoses. In addition to using NGS of ctDNA for the detection of clonal Ig gene rearrangements to help prioritize PLWH for lymphoma diagnosis, several studies have evaluated this approach for early detection of relapsed or refractory disease.^38,39^ Although more research is needed to better understand the kinetics of ctDNA during various lymphoma therapies, the idea of monitoring treatment response in blood would be desirable in a resource-limited setting, where radiologic evaluation of treatment response with positron emission tomography/computed tomography and traditional computed tomography is limited.

NGS requires a relatively modest investment in research to validate the approach. Thus, the NGS analysis of a plasma specimen or lymph node aspirate might facilitate efficient prioritization of patients for biopsy to establish a diagnosis of lymphoma and accelerate the initiation of curative therapy.
